# Dynamic multilayer growth: Parallel vs. sequential approaches

**DOI:** 10.1371/journal.pone.0301513

**Published:** 2024-05-09

**Authors:** Matt Ross, Nareg Berberian, Albino Nikolla, Sylvain Chartier

**Affiliations:** Laboratory for- Computational Neurodynamics and Cognition, School of Psychology, University of Ottawa, Ottawa, ON, Canada; Vrije Universiteit Amsterdam, NETHERLANDS

## Abstract

The decision of when to add a new hidden unit or layer is a fundamental challenge for constructive algorithms. It becomes even more complex in the context of multiple hidden layers. Growing both network width and depth offers a robust framework for leveraging the ability to capture more information from the data and model more complex representations. In the context of multiple hidden layers, should growing units occur sequentially with hidden units only being grown in one layer at a time or in parallel with hidden units growing across multiple layers simultaneously? The effects of growing sequentially or in parallel are investigated using a population dynamics-inspired growing algorithm in a multilayer context. A modified version of the constructive growing algorithm capable of growing in parallel is presented. Sequential and parallel growth methodologies are compared in a three-hidden layer multilayer perceptron on several benchmark classification tasks. Several variants of these approaches are developed for a more in-depth comparison based on the type of hidden layer initialization and the weight update methods employed. Comparisons are then made to another sequential growing approach, Dynamic Node Creation. Growing hidden layers in parallel resulted in comparable or higher performances than sequential approaches. Growing hidden layers in parallel promotes growing narrower deep architectures tailored to the task. Dynamic growth inspired by population dynamics offers the potential to grow the width and depth of deeper neural networks in either a sequential or parallel fashion.

## Introduction

When deciding the architecture of an artificial neural network, a fundamental question arises; is it better to use a shallow and wide network or deep and narrow? This problem is commonly known as the trade-off between width and depth. Where width refers to the number of units in a hidden layer and depth refers to the number of hidden and output layers [1].

Previously, it was shown that multilayer feedforward networks with a single hidden layer and a large enough width could approximate any continuous function, making these shallow networks universal approximators [2,3]. Despite this, the ability of deeper architectures to learn more complex, distributed, and sparse representations make them more powerful than their shallow counterparts [1,4–6]. With the increase in network depth, learning more abstract and complex representations can occur, allowing the network to discriminate inputs better [5,7]. This brings us back to the trade-off, is depth more valuable than width? Bianchini and Scarselli [4] used Betti numbers, a topological measure, to compare shallow and deep feedforward networks. They showed that a deep neural network with the same number of hidden units as its shallow counterpart could realize more complex functions. Eldan and Shamir [6] demonstrated that for a fully connected feedforward network with a linear-output unit, “depth-even if increased by 1- can be exponentially more valuable than width for standard feedforward networks.” With larger data sets and more powerful graphics processing units, the increased representational power and faster training of deep neural networks have made them valuable tools. Deep neural networks have been applied to many different problems, including computer vision, object detection, and electroencephalogram classification, to name a few (for reviews, see [5,8–10]).

A network’s depth and width affect the neural network’s ability to generalize. If the network architecture is too large, the model may overfit the data and cause poor generalization. Conversely, if the network architecture is too small, the model may underfit the data and cause over-generalization [11–15]. This reinforces the need to find optimal architectures that match a given task complexity. Currently, the best practice is to use fixed neural architectures found using a trial-and-error approach. This is due to its simplicity of implementation. However, this process can be temporally cumbersome and there is no guarantee that an optimal or near-optimal topology will be found [11,14,16,17].

An alternative to using fixed architectures found by a trial-and-error approach is to use adaptive neural architectures. With adaptive neural architectures, the idea is to have a dynamic architecture that is updated during training [18]. Several strategies have been proposed and can be broadly classified into three categories: constructive algorithms, pruning algorithms, and hybrid methods.

Constructive algorithms involve starting with a small network architecture (typically one hidden unit) and gradually adding connections, units, or layers during training to match task complexity [18–20]. Conversely, pruning algorithms involve starting with a larger network where connections and units are pruned (removed) during the learning process to match task complexity (for surveys, see [21–24]). Hybrid methods combine constructive and pruning algorithms. These methods typically involve using a constructive algorithm to grow the neural architecture first, then prune the subsequent architecture, or simultaneously grow and prune during the learning process [25]. While using a hybrid approach is appealing and has had great success [16,26–31]; the focus of the present article is on constructive algorithms.

Constructive algorithms offer the possibility of compact architectures as an alternative to the trial-and-error approach when designing architectures. The focus of constructive algorithms has primarily been on growing the width of a single hidden layer. Examples of applications with this focus include regression problems [32,33], classification [12,13,20,34–46], and image segmentation [47] to name a few (for a list of more applications, see [19]). Conversely, approaches based on cascade correlation have offered a constructive approach that focused on growing network depth instead of width. These approaches use a cascaded architecture where the network grows many hidden layers the width of a single unit [31,48–52].

Growing both network width and depth offers a robust framework for leveraging the ability to capture more information from the data and model more complex representations. There have been numerous approaches for determining when new hidden units and layers should be added [14,53–57]. For instance, a measure of network significance to quantify generalization power has been proposed for growing network width in combination with drift detection for growing depth [58,59]. Others have examined whether the error or loss has stopped changing by a predetermined threshold value. Baluja and Fahlman [60] proposed a sibling/descendant cascade correlation, where once the error stops changing, candidate units can be added to the same layer (siblings) or a new layer (descendant). The candidate pool is made of both types of units, and whichever unit reduces the residual network error the most is added to the network. Zemouri et al., [16] introduced the growing pruning deep learning neural network (GP-DLNN) that uses a penalty term to and compared to a threshold value. If the penalty is smaller than the threshold, a new unit is added, and a new layer is added if larger. Similarly, the cascade-correlation growing deep learning neural network CCG-DLNN, a constructive approach grounded in cascade correlation, also used the same approach to decide if candidate units should be added to the more recent layer or a new layer [29]. Another approach is to set the maximum number of hidden units and layers in advance, as with the evolutionary algorithm for building deep neural networks [17,61]. This approach calculates and compares the error at each step to a threshold value. Growth is complete if the error converges or the maximum allowed structure is reached. Only if the error convergence is not reached and the maximum number of hidden units for that layer is reached will a new layer be added [17,61].

When employing constructive algorithms, serval challenges have been identified and should be considered [16,25]:

- What are the criteria for adding a new unit or layer to the network?
- How to connect a newly added hidden unit?
- How should the weights be initialized?
- What training scheme should be used (re-train the whole network vs. freezing)?
- When to stop adding hidden units (what are the convergence criteria)?

Here, we focus on the first challenge: the criteria for adding a new unit or layer to the network. Specifically, we are interested in *when a new hidden unit or layer should be added*. In the context of multiple hidden layers, should growing units occur sequentially or in parallel across hidden layers? With a sequential growth methodology, we refer to only being able to grow hidden units in one hidden layer at a time. The examples of constructive algorithms that grow both width and depth mentioned previously fall under this methodology. Contrarily, with a parallel growth methodology, we refer to being able to grow hidden units across multiple layers simultaneously. This methodology is scarcely found in the literature, with a modular network approach being the most evident. Guan and Li [62] used such an approach. They broke benchmark classification problems into simpler subproblems using task decomposition and output parallelism. Modules were then grown and trained in parallel to solve each subproblem. The resulting modules that could solve the subproblems were merged into a modular network.

Previously, we introduced a constructive growing algorithm that provides a more self-governed alternative for deciding when a new unit should be added to a single hidden layer multilayer perceptron [63]. This approach was inspired by population dynamics [64] and considered the hidden units as a population and the hidden layer as the environment they exist in. This endowed the hidden layer with a carrying capacity, the maximum population the hidden layer environment can sustain (an upper bound on the layer width). This allowed the application of population dynamics to provide a hidden unit population growth rate for the hidden layer. Combining the carrying capacity and direct performance feedback from the network created a built-in dynamic, a more self-governed method for growing the hidden layer, simultaneously preventing growing outbound. A natural extension of this work would be to grow both width and depth. Since each hidden layer is treated as having its hidden unit population, should the growth rate of each population be considered individually in a sequential fashion or simultaneously in a parallel fashion?

We propose investigating the effects of growing sequentially or in parallel using our population dynamics inspired growing algorithm in a multilayer context. To achieve this, we create a modified version of our constructive growing algorithm capable of growing in parallel. We then test the methodologies of sequential and parallel growth in a three-hidden layer multilayer perceptron on several benchmark classification tasks. We test several variants of these approaches for a more intricate comparison based on the hidden layer initialization and the training scheme. Comparisons are also made to another sequential growing approach, Dynamic Node Creation (DNC; [35]), that employs the common methodology of iteratively adding units based on the error curve flattening within a specific time frame.

The remainder of the paper is divided as follows: Section II introduces the growing approaches employed (sequential growth, parallel growth, DNC growth), the network’s architecture and variants, and the learning procedure. In Section III, we describe the results of several benchmark classification tasks. Finally, in Sections IV and V, we discuss and conclude the study’s findings.

## Methods

### Growing

#### Sequential growth

**Fig 1:**
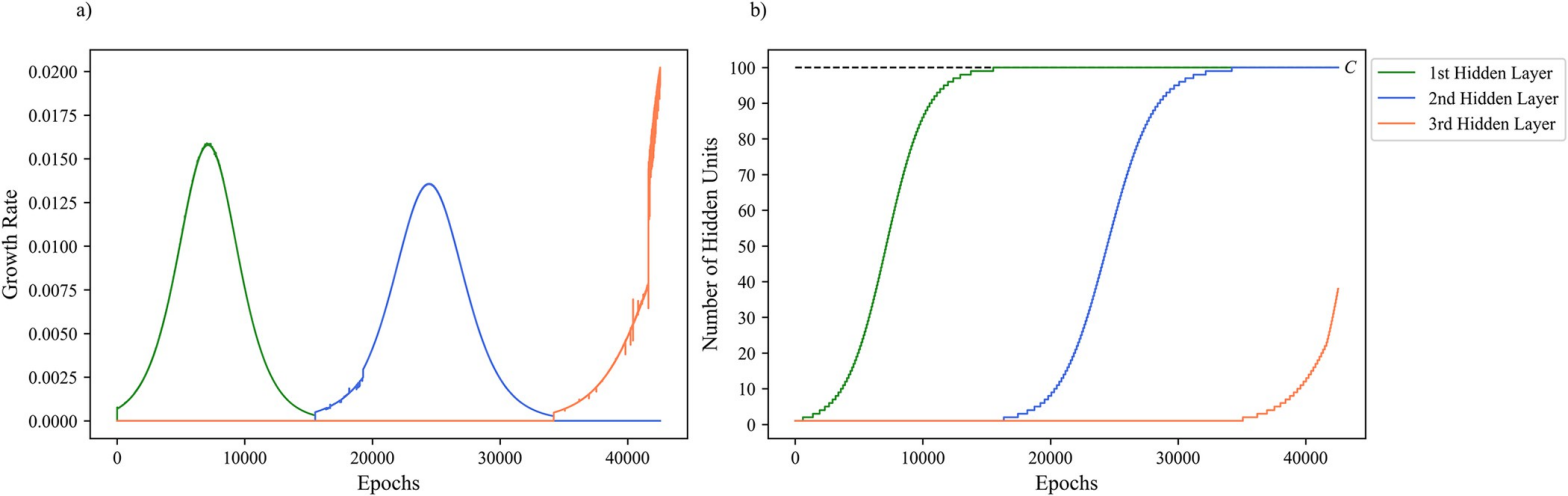
A sample of sequential growing dynamics for a three-hidden layer MLP. a) Sequential growth rate of three-hidden layers across training epochs. b) Changes in hidden layer size across three-hidden layers with a carrying capacity (*C*) of 100 during training.

The dynamics of the sequential growing algorithm are characterized by having only one hidden layer able to grow at a time. In this context, the network is not able to grow the subsequent layer (ℓ+1) until the current layer (ℓ) has reached the maximum number of hidden units that it can sustain as dictated by the carrying capacity (*C*). A visualization of the sequential growing method’s effect on growth rate and changes in hidden layer sizes for a three-hidden layer MLP is depicted in Fig 1.

Sequential growth of the hidden layers is dictated by Eq (1). This algorithm was inspired by the Single-species model with the Allee effect [64]. It is used to calculate the change in the size of the hidden unit population (i.e., growth rate; $\frac{dh_{s}}{dt}$) for each hidden layer (ℓ). Incorporated into the algorithm is the global network error (*E*) as calculated by the loss function. This allows the network’s performance to modulate the growth rate of the hidden unit population during learning. The result is that regardless of what the carrying capacity (*C*) is set to, the hidden unit population will grow based on the needs of the network. As such the carrying capacity is defined as *C*∈ℕ_1_|*C*<∞. The addition of a new unit to the hidden layer only occurs when the growth rate reaches an integer value, as adding a proportion of a single unit is not plausible. The extinction constant (*α*) acts as a lower bound on the size of the hidden layer population. The population will go locally extinct and prune units if the population falls below this threshold value. As this study aims to examine growth, *α* is set just below zero to remove the possibility of extinction.

$$\frac{dh_{s}^{l}}{dt}=\frac{1}{E+l}*\left(\frac{1}{C}+1\right)*E*\left(1-\frac{h_{s}^{l}}{C}\right)(h_{s}^{l}-\alpha )$$ (1)

where *C* is the carrying capacity of the hidden layer environment (upper bound), ℓ is the hidden layer number, *h*_*s*_ is the growth of the hidden unit population, *E* is the current global error of the network, and *α* is the extinction constant (lower bound) set to -0.1.

#### Parallel growth

**Fig 2:**
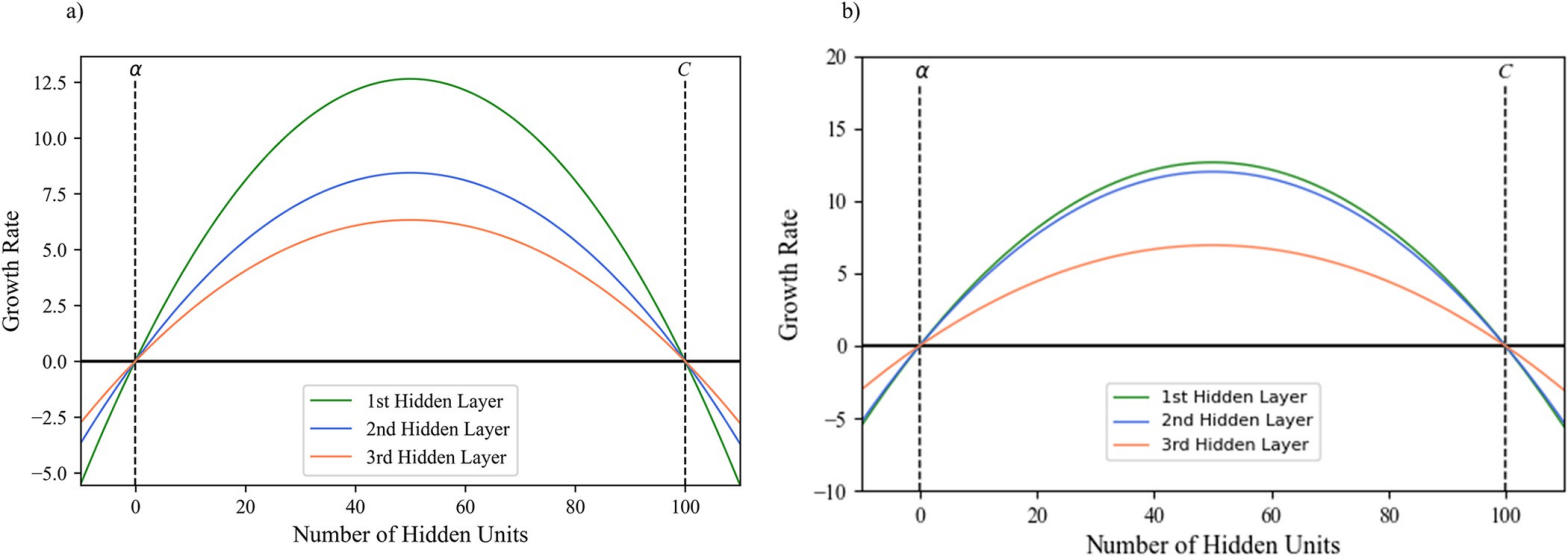
The effect of hidden layer size on the growth rate of subsequent layers. Where *C* is the carrying capacity and *α* is the extinction constant. a) Initial staggered growth rate of hidden layers, with deeper layers growing at a slower rate. b) Increase in the second layers growth rate as a response to the first hidden layer almost reaching the carrying capacity.

**Fig 3:**
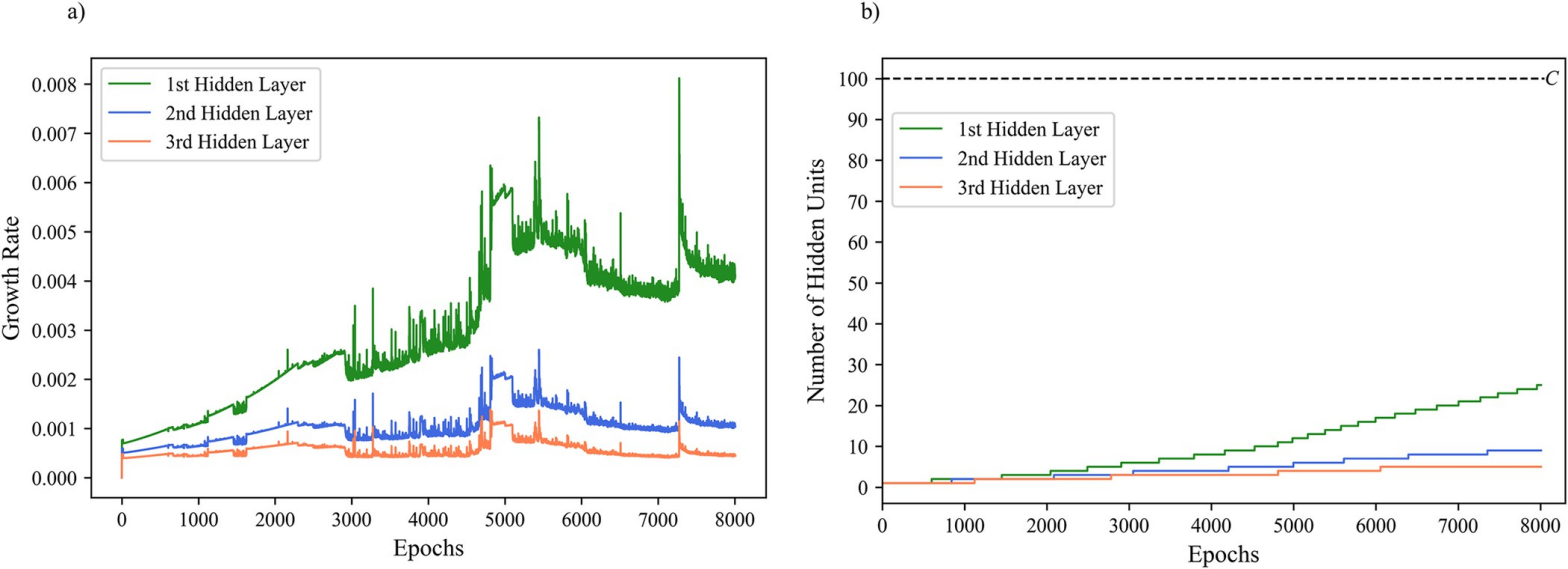
A sample of parallel growing dynamics for a three-hidden layer MLP. a) Parallel growth rate of three-hidden layers across training epochs. b) Changes in hidden layer size across three-hidden layers with a carrying capacity (*C*) of 100 during training.

The dynamics of parallel growing algorithm are characterized by having all hidden layers in the network growing simultaneously. In this context, the network can grow the current layer (ℓ) and all subsequent layers (ℓ⋯*m*) regardless if the carrying capacity (*C*) has been reached by any hidden layer. The growth rate of the first hidden layer $\left(h_{s}^{0}\right)$ is calculated according to Eq (1), while the growth rate of every subsequent layer ($h_{s}^{l}$) is calculated according to Eq (2). The growth rate of the first layer is independent of the size of any other layer, in contract subsequent hidden layers are impacted by the size of the hidden layer that proceeds it ($h_{s}^{l-1}$). The effect of previous hidden layer sizes being considered allows for a more staggered growth rate for deeper layers. As the previous hidden layer increases in size and begins to approach the carrying capacity, where once it reached it will no longer be able to add any new hidden units, the current layers growth rate is dynamically increased to compensate for the continued need to add more hidden units to match the tasks complexity (see Fig 2). A visualization of the parallel growing method’s effect on growth rate and changes in hidden layer sizes for a three-hidden layer MLP is depicted in Fig 3.

$$\frac{dh_{s}^{l}}{dt}=\frac{1}{E+l}*\left(\frac{1}{C-h_{s}^{l-1}}+1\right)*E*\left(1-\frac{h_{s}^{l}}{C}\right)(h_{s}^{l}-\alpha )$$ (2)

where *C* is the carrying capacity of the hidden layer environment (upper bound), $h_{s}^{l}$ is the growth of the hidden unit population of the current layer, $h_{s}^{l-1}$ is the growth of the hidden unit population of the previous layer, E is the current global error of the network, and *α* is the extinction constant (lower bound) set to -0.1.

#### DNC growth

Iteratively adding new units with DNC occurs when the error curve flattens within a specific time frame (i.e., window width; [35]). This requires an *a priori* finely-tuned trigger slope (Δ_*T*_), which measures the error curve’s flatness, whereby a new unit is added if the error falls below this value. Additionally, the user must finely tune the window width (*z*), the number of epochs over which the flatness of the error curve is compared to the trigger slope. If the error curve flattens out below the trigger slope (Δ_*T*_), and if enough epochs have passed since the last addition, a new unit is added. This is determined by Eqs (3) and (4). The window width (*z*) was set to 1000 epochs and the trigger slope (Δ_*T*_) was set to 0.01 to promote the construction of smaller architectures. To allow a comparison in the context of multiple hidden layers, here, DNC follows a sequential growth methodology. The network is not able to grow the subsequent layer (ℓ+1) until the current layer (ℓ) has reached the maximum number of hidden units that it can sustain as dictated by the carrying capacity (*C*).

$$\frac{\rho_{\left[t\right]}-\rho_{\left[t-z\right]}}{\rho_{{[t}_{0]}}}<\Delta_{T}$$ (3)

where *ρ*_[*t*]_ is the average error at time *t* across all output units, *z* is the window width in epochs over which the slope is determined, Δ_*T*_ is the trigger slope.

$$t-z\geq t_{0}$$ (4)

where *t*_0_ is the time in epochs when the last unit was added to the hidden layer.

### Architecture: A formal description

**Fig 4:**
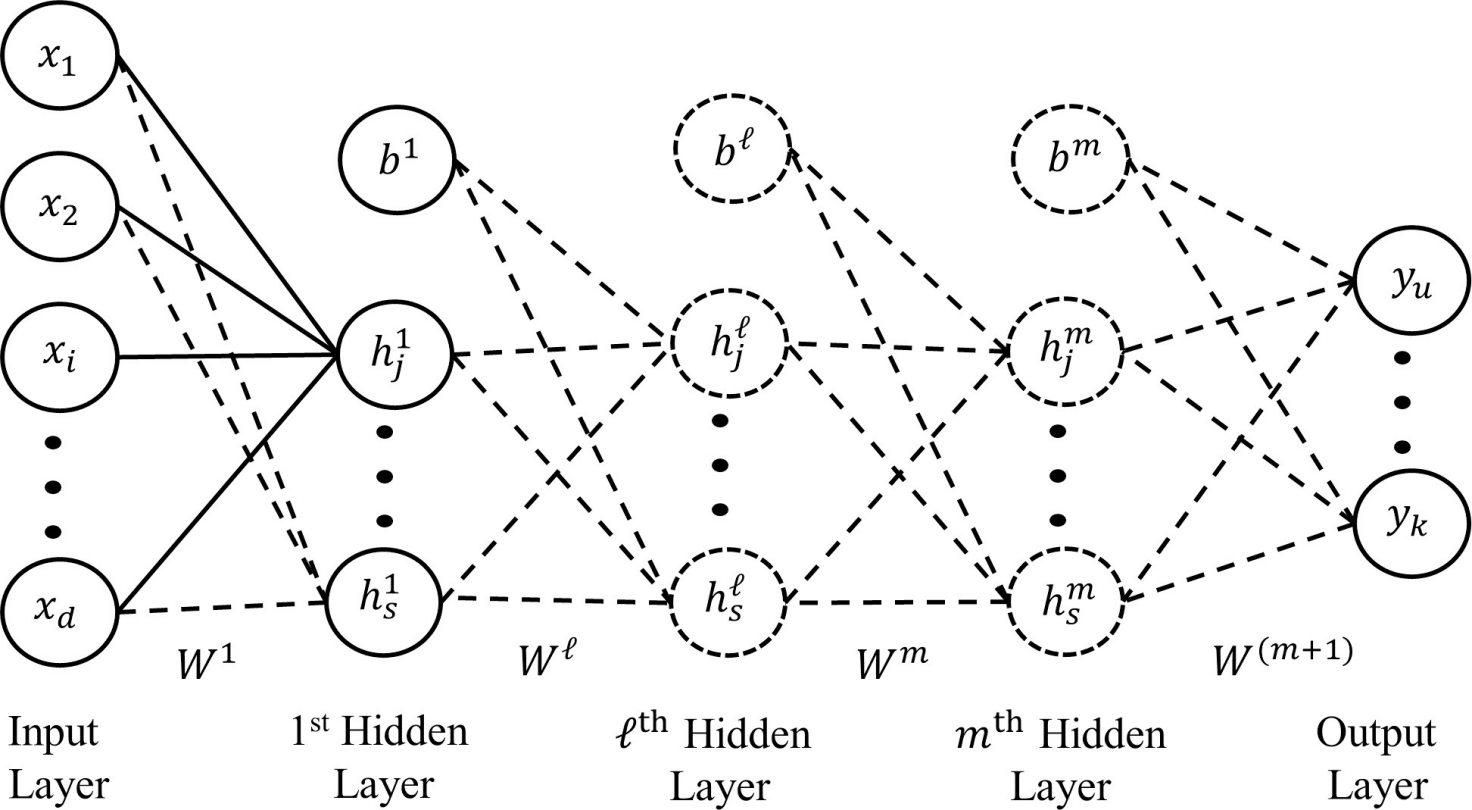
MLP architecture. In the above architecture x is vector of length *d*, where *d*, is the dimension the input vector, *s* is the current number of hidden units in that layer, *b* the biases, and the dashed lines and dashed circles are weight connections and hidden units that are incrementally added during growing.

A multilayer perceptron (MLP) with *m* hidden layers is described by the number of units and the number of weight connections (see Fig 4).

Let the size of layer ℓ at training epoch *t* be denoted by $S_{\left[t\right]}^{0}$, with the size of the all the layers in the network at training epoch *t* be defined by vector *ψ*_[*t*]_. The architecture of the network is thus represented as $\psi_{\left[t\right]}=(S_{\left[t\right]}^{0},\;S_{\left[t\right]}^{1},\;S_{\left[t\right]}^{2},\;\cdots S_{\left[t\right]}^{m},\;\cdots S_{\left[t\right]}^{m+1})$. With *m* being the total number of hidden layers, $S_{\left[t\right]}^{0}$ being the size of the input layer (*d*), and $S_{\left[t\right]}^{m+1}$ the size of the output layer (*k*). The weights for each layer are defined by tensor *Φ*. With *Φ* = (*W*^1^,*W*^2^,⋯*W*^*m*^⋯,*W*^(*m*+1)^). Wherein each component *W*^ℓ^ of this tensor is a weight connection matrix:

$$W^{l}=\left(\begin{array}{ccccc} w_{11}^{l} & \cdots & w_{1j}^{l} & \cdots & w_{1S_{\left(l-1\right)}}^{l}\\ \vdots & \ddots & \vdots & \ddots & \vdots \\ w_{i1}^{l} & \cdots & w_{ij}^{l} & \cdots & w_{iS_{\left(l-1\right)}}^{l}\\ \vdots & \ddots & \vdots & \ddots & \vdots \\ w_{S_{l}1}^{l} & \cdots & w_{S_{l}j}^{l} & \cdots & w_{S_{l}S_{\left(l-1\right)}}^{l} \end{array}\right)$$

where $w_{ij}^{l}$ is the weight connection between the *i*^*th*^ unit of the layer ℓ and the *j*^*th*^ unit of layer ℓ−1.

The size of the input layer (*d*) and output layer (*k*) are fixed and dependent on the given task. In contrast, the width and depth of the hidden layers (*ψ*_[*t*]_) are permitted to grow. For all variants the maximum number of hidden layers is defined by *Max*_ℓ_ and the maximum number of hidden units per hidden layer is dictated by the carrying capacity (*C*). To simplify comparisons, *Max*_ℓ_ was fixed to three hidden layers, and *C* was fixed at 100 hidden units. The initialization condition gives rise to different network variants.

**Fig 5:**
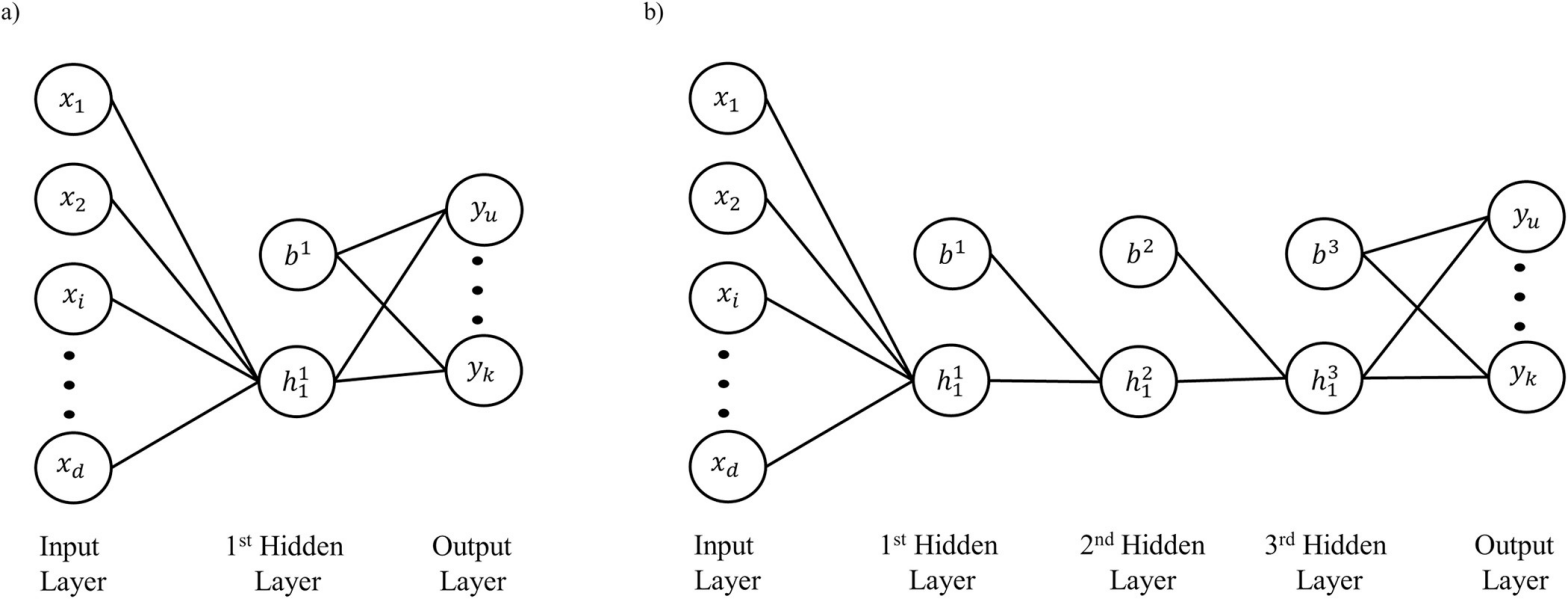
Network initialization conditions. a) Only one hidden layer initialized with a single hidden unit. b) All three hidden layers initialized with a single hidden unit.

- In the first condition, the network is only initialized with one hidden layer with a single hidden unit (First-Init variant), $\psi_{\left[0\right]}=(S_{\left[0\right]}^{0},\;S_{\left[0\right]}^{1},\;S_{\left[0\right]}^{2})=(d,1,k)$, for a visualization see Fig 5A.
- Alternatively, the second condition initializes all three hidden layers with a single hidden unit (All-Init variant), $\psi_{\left[0\right]}=(S_{\left[0\right]}^{0},\;S_{\left[0\right]}^{1},\;S_{\left[0\right]}^{2},\;S_{\left[0\right]}^{3},\;S_{\left[0\right]}^{4})=(d,\;1,\;1,\;1,\;k)$, for a visualization see Fig 5B.

**Fig 6:**
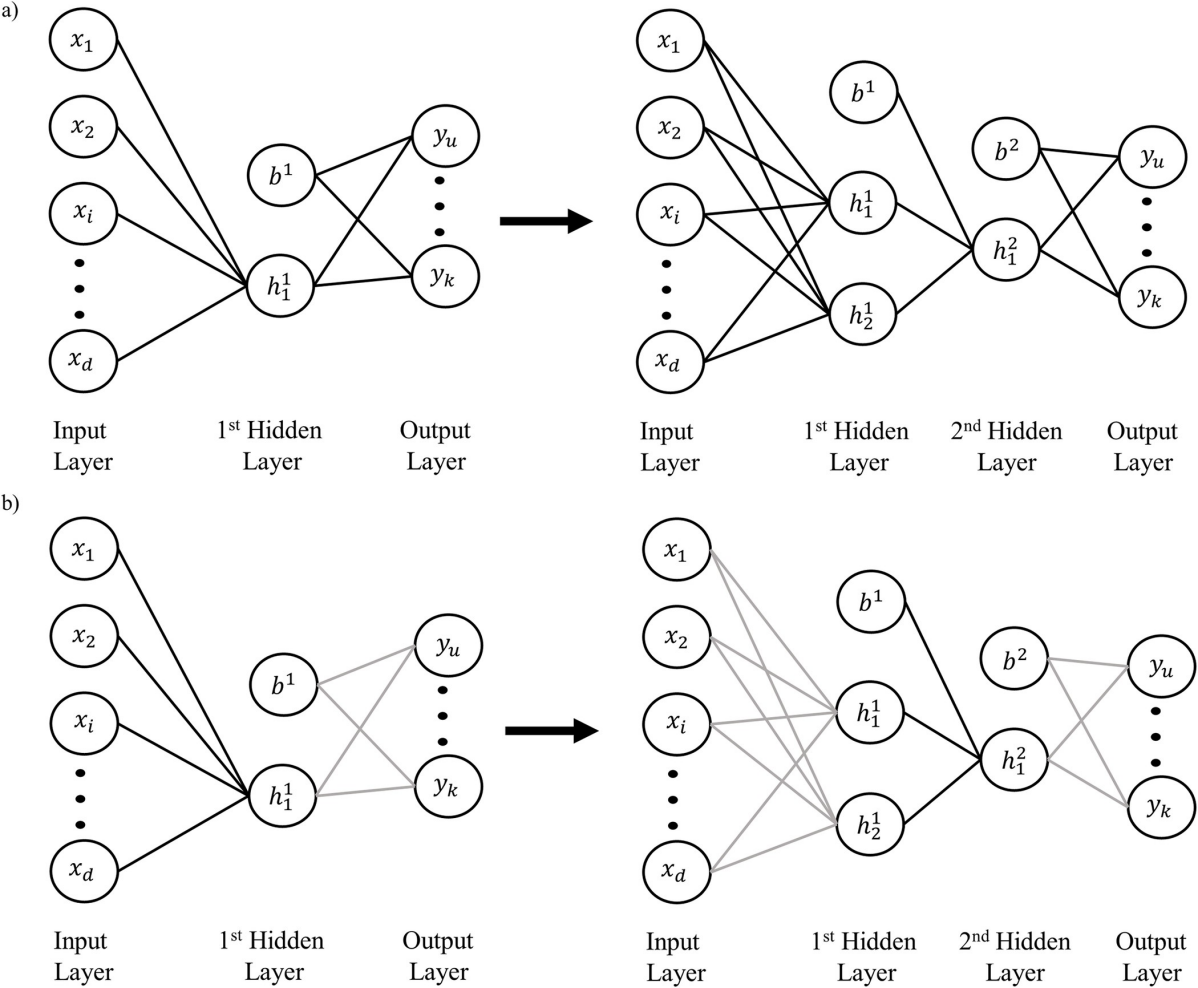
Weight update conditions. a) All network weights can be updated as the network grows (solid black lines). b) Only the incoming weights to the layer that is actively growing can be updated (solid black lines), all other weights in the network are frozen (light grey lines). In this example, initially the first hidden layer is active, then second hidden layer grows a new unit and becomes the active layer.

When adding a new unit with any of the growing conditions (sequential, parallel, or DNC), the new unit is added to the end of the hidden layer with all associated incoming and outgoing weight connections in a fully connected fashion. These weight connections are randomly initialized. During learning, the weights are updated via batch stochastic gradient descent with momentum by two possible conditions:

- All weights in the network are updated at each training epoch *t* (UAW variant), for a visualization see Fig 6A.
- Only the incoming weights to the layer that is actively growing are updated and all other weights in the network are frozen (F variant), for a visualization see Fig 6B.

### Learning

The following section outlines the learning process and the algorithms for each of the growing approaches. To measure network performance, the categorical cross entropy loss function (5) is calculated at the end of each epoch as a measure of error with the addition of a weight penalty from *L*_2_ regularization (ridge regression).

$$E_{\left[t\right]}=\left(-\frac{1}{M}\sum_{k=1}^{N}\sum_{q=1}^{M}y_{k}^{q}\log{\hat{y}}_{k}^{q}\right)+\left(\frac{\lambda }{2M}\sum_{i=1}^{d}{W_{\left[t\right]}^{i}}^{2}\right)$$ (5)

where ${\hat{y}}_{k}^{q}$ is the *k*^*th*^ scalar value output from the network for example *q*, $y_{k}^{q}$ is the corresponding target value, and *N* is the total number of classes, and *M* is the total number of instances in the data, *d* is the number of input features, and *λ* the regularization parameter tuned to 0.001.

At each training epoch *t*,*R* batch iterations of batch stochastic gradient descent with momentum are computed to update the weights *W*^ℓ^. At each batch iteration *iter*(*iter* = 1 to *R*), momentum, the exponential moving average, is calculated according to:

$$m_{\left[t\right]}=\beta m_{\left[t-1\right]}+(1-\beta )g_{\left[t\right]}$$ (6)

where *m*_[*t*]_ is the velocity vector, *β* is the momentum term (friction coefficient) set at 0.9, and *g*_[*t*]_ is the gradient at time *t*.

The weights are then updated according to:

$$W_{\left[t\right]}^{l}=W_{\left[t-1\right]}^{l}-\eta (m_{\left[t\right]})$$ (7)

where *η* is the learning rate set to 0.01.

The sigmoid activation function (8) is used, with the softmax function (9) being used to scale the final output of the network and generate interrelated probabilities for multiclass classification between 0 and 1.

$$h_{j}^{l}=\frac{1}{1+e^{-(h_{j}^{lin})}}$$ (8)

where $h_{j}^{l}$ is the hidden layer output from unit *j* for layer ℓ obtained in the usual way (for details, see [63]) and $h_{j}^{lin}$ is its corresponding activation.

$$\hat{y}=\frac{e^{y_{u}^{in}}}{\sum_{k=1}^{N}e^{y_{k}^{in}}}$$ (9)

where $\hat{y}$ is the categorical probabilistic output vector that sums to 1 and $y_{u}^{in}$ is the input vector. The denominator is a normalization term across all classes (*N*).

With all approaches, training continues until one of the following conditions are met:

1. The maximum number of training epochs is reached, *Max*_*t*_ = 100,000.
2. The training categorical cross entropy falls below the convergence threshold, *Min*_*E*_ = 0.01.
3. The difference in testing accuracy over a 1000 epoch window is less than the testing accuracy threshold, *θ* = 0.01, and the testing accuracy from the last epoch is >0.8 (80%).

### Growing algorithms

As previously explained in the architecture section, four potential network variants for each growing approach are explored. Consequently, each algorithm is composed of two essential components: its initialization condition (referred to as All Init-Variant or Part 1a, and First Init-Variant or Part 1b) and its weight update condition (referred to as UAW-Variant or Part 2a, and the F-Variant Part 2b). The specific details of the growing algorithms for both the parallel and sequential approaches are outlined below.

### Parallel growth

**Part 1a Initialization: All Init-Variant**

*//Initialize all hidden layers*.

*t = 0*, Starting epoch.

*Max*_ℓ_ = 3, Maximum number of hidden layers.

*Init*_ℓ_ = 3, Initial number of hidden layers.

*C* = 100, Maximum number of hidden units that a layer can sustain.

*if* F-Variant*//Only the incoming weights to active layer are updated and others frozen*:

*Activ*_ℓ_ = 1, Initial active layer.

*//Initialization of the network topology*:

$\psi_{\left[t\right]}=(S_{\left[0\right]}^{0}+S_{\left[0\right]}^{1}+S_{\left[0\right]}^{2}+S_{\left[0\right]}^{3}+S_{\left[0\right]}^{4})=(d,\;1,\;1,\;1,\;y)$

*//Random initialization of the weights from a uniform distribution ranging from -0*.*1 to 0*.*1*:

$\Phi =(W^{1}+W^{2}+W^{3}+W^{4})$

*// Initialization of the growth rate for the hidden layers*:

$\varrho_{\left[t\right]}=(h_{s[0]}^{1}+h_{s[0]}^{2}+h_{s[0]}^{3})=(0,\;0,\;0)$

**Part 1b Initialization: First Init-Variant**

//Initialize only the first hidden layer.

 *t* = 0, Starting epoch.

*Max*_ℓ_ = 3, Maximum number of hidden layers.

*Init*_ℓ_ = 1, Initial number of hidden layers.

*C* = 100, Maximum number of hidden units that a layer can sustain.

if F-Variant//Only the incoming weights to active layer are updated and others frozen:

*Activ*_ℓ_ = 1 Initial active layer.

//Initialization of the network topology:

$\psi_{\left[t\right]}=(S_{\left[0\right]}^{0}+S_{\left[0\right]}^{1}+S_{\left[0\right]}^{2})=(d,\;1,\;y)$

//Random initialization of the weights from a uniform distribution ranging from -0.1 to 0.1:

$\Phi =(W^{1}+W^{2})$

// Initialization of the growth rate for the hidden layers:

$\varrho_{\left[t\right]}=(h_{s[0]}^{1}+h_{s[0]}^{2}+h_{s[0]}^{3})=(0,\;0,\;0)$

**Part 2a Learning and Parallel Growing: UAW-Variant**

*//Update all weights*.

*done = false*

$While\;t<{Max}_{t}\;and\;done=false$

*for iter* = *1 to R*

calculate $m_{\left[t\right]}=\beta m_{\left[t-1\right]}+(1-\beta )g_{\left[t\right]}$

calculate $W_{\left[t\right]}^{l}=W_{\left[t-1\right]}^{l}-\eta (m_{\left[t\right]})$

*end iter*

       calculate loss

$E_{\left[t\right]}=\left(-\frac{1}{M}\sum_{k=1}^{N}\sum_{q=1}^{M}y_{q}^{k}\log{\hat{y}}_{q}^{k}\right)+\left(\frac{\lambda }{2M}\sum_{i=1}^{n}{W_{\left[t\right]}^{i}}^{2}\right)$

       calculate accuracy *TrainAcc*_[*t*]_ and *TestAcc*_[t]_

       calculate growth rate

      for ℓ = 1 to *m*

     *if ℓ = 1*

    $\frac{dh_{s}^{l}}{dt}=\frac{1}{E+l}*(\frac{1}{C}+1)*E*(1-\frac{h_{s}^{l}}{C})(h_{s}^{l}-\alpha )$

          *else*

$\frac{dh_{s}^{l}}{dt}=\frac{1}{E+l}*(\frac{1}{C-h_{s}^{l-1}}+1)*E*(1-\frac{h_{s}^{l}}{C})(h_{s}^{l}-\alpha )$

*//Update the growth rate*

$h_{s[t]}^{l}=h_{s[t-1]}^{l}+\frac{dh_{s}^{l}}{dt}$

          *//Determine if a new unit is grown and has not reached the carrying capacity*.

                if $h_{s[t]}^{l}-h_{s[t-1]}^{l}=1\;and\;h_{s[t]}^{l}<C$

 *// A new hidden unit has been grown*.

                    $S_{\left[t\right]}^{l}=S_{\left[t-1\right]}^{l}+1$

                      *//Random initialization of the new unit’s weights from a uniform distribution ranging from -0*.*1 to 0*.*1*.

      if *E*_[*t*]_<*Min*_*E*_ or t = *Max*_*t*_ or (*TestAcc*_[*t*]_−*TextAcc*_[*t*−1000]_)<θ

*done* = *true*

 *else*

*t = t+1*

**Part 2b Learning and Parallel Growing: F-Variant**

*//Only the incoming weights to active layer are updated and others frozen*.

*done = false*

$While\;t<{Max}_{t}\;and\;done=false$

*for iter = 1 to R*

calculate *m*[*t*] = *βm*_[*t*−1]_+(1−*β*)*g*_[*t*]_

*//Only the incoming weights to the active layer are updated*.

calculate $W_{\left[t\right]}^{{Activ}_{l}}=W_{\left[t-1\right]}^{{Activ}_{l}}-\eta (m_{\left[t\right]})$

*end iter*

       calculate loss

$E_{\left[t\right]}=\left(-\frac{1}{M}\sum_{k=1}^{N}\sum_{q=1}^{M}y_{q}^{k}\log{\hat{y}}_{q}^{k}\right)+\left(\frac{\lambda }{2M}\sum_{i=1}^{n}{W_{\left[t\right]}^{i}}^{2}\right)$

       calculate accuracy *TrainAcc*_[*t*]_ and *TestAcc*_[*t*]_

       calculate growth rate

      *for* ℓ = 1 to *m*

     *if ℓ = 1*

    $\frac{dh_{s}^{l}}{dt}=\frac{1}{E+l}*(\frac{1}{C}+1)*E*(1-\frac{h_{s}^{l}}{C})(h_{s}^{l}-\alpha )$

          *else*

$\frac{dh_{s}^{l}}{dt}=\frac{1}{E+l}*(\frac{1}{C-h_{s}^{l-1}}+1)*E*(1-\frac{h_{s}^{l}}{C})(h_{s}^{l}-\alpha )$

*//Update the growth rate*

$h_{s[t]}^{l}=h_{s[t-1]}^{l}+\frac{dh_{s}^{l}}{dt}$

          *//Determine if a new unit is grown and has not reached the carrying capacity*.

                if $h_{s[t]}^{l}-h_{s[t-1]}^{l}=1\;and\;h_{s[t]}^{l}<C$

 *// A new hidden unit has been grown*.

                    $S_{\left[t\right]}^{l}=S_{\left[t-1\right]}^{l}+1$

                     *//Random initialization of the new unit’s weights from a uniform distribution ranging from -0*.*1 to 0*.*1*.

*// Has the new unit been grown in a different layer than the active layer*?

*if ℓ*≠*Activ*_ℓ_

                 *Activ*_ℓ_ = ℓ *//Update which is the active layer*.

      if *E*_[*t*]_<*Min*_*E*_ or *t = Max*_*t*_ or (*TestAcc*_[*t*]_−*TestAcc*_[*t*−1000]_)<*θ*

*done* = *true*

 *else*

*t* = *t+1*

### Sequential growth and DNC growth

**Part 1a Initialization: All Init-Variant**

*//Initialize all hidden layers*.

 *t* = 0, Starting epoch.

*Max*_ℓ_ = 3, Maximum number of hidden layers.

*Init*_ℓ_ = 3, Initial number of hidden layers.

*Current*_ℓ_ = 1, Starting layer for sequential growth.

*C* = 100, Maximum number of hidden units that a layer can sustain.

*//Initialization of the network topology*:

$\psi_{\left[t\right]}=(S_{\left[0\right]}^{0}+S_{\left[0\right]}^{1}+S_{\left[0\right]}^{2}+S_{\left[0\right]}^{3}+S_{\left[0\right]}^{4})=(d,\;1,\;1,\;1,\;y)$

*//Random initialization of the weights from a uniform distribution ranging from -0*.*1 to 0*.*1*:

$\Phi =(W^{1}+W^{2}+W^{3}+W^{4})$

*if* Sequential:

 *// Initialization of the growth rate for the hidden layers*:

$\varrho_{\left[t\right]}=(h_{s[0]}^{1}+h_{s[0]}^{2}+h_{s[0]}^{3})=(0,\;0,\;0)$

**Part 1b Initialization: First Init-Variant**

*//Initialize only the first hidden layer*.

*t = 0*, Starting epoch.

*Max*_ℓ_ = 3, Maximum number of hidden layers.

*Init*_ℓ_ = 1, Initial number of hidden layers.

*Current*_ℓ_ = 1, Starting layer for sequential growth.

*C* = 100, Maximum number of hidden units that a layer can sustain.

*//Initialization of the network topology*:

$\psi_{\left[t\right]}=(S_{\left[0\right]}^{0}+S_{\left[0\right]}^{1}+S_{\left[0\right]}^{2})=(d,\;1,\;y)$

*//Random initialization of the weights from a uniform distribution ranging from -0*.*1 to 0*.*1*:

$\Phi =(W^{1}+W^{2})$

*if* Sequential growth:

*// Initialization of the growth rate for the hidden layers*:

$\varrho_{\left[t\right]}=(h_{s[0]}^{1}+h_{s[0]}^{2}+h_{s[0]}^{3})=(0,\;0,\;0)$

1) Sequential growth:

**Part 2a Learning and Sequential Growing: UAW-Variant**

 *//Update all weights*.

*done = false*

$While\;t<{Max}_{t}\;and\;done=false$

*for iter = 1 to R*

calculate *m*_[*t*]_ = *βm*_[*t*−1]_+(1−*β*)*g*_[*t*]_

calculate $W_{\left[t\right]}^{l}=W_{\left[t-1\right]}^{l}-\eta (m_{\left[t\right]})$

*end iter*

       calculate loss

$E_{\left[t\right]}=\left(-\frac{1}{M}\sum_{k=1}^{N}\sum_{q=1}^{M}y_{q}^{k}\log{\hat{y}}_{q}^{k}\right)+\left(\frac{\lambda }{2M}\sum_{i=1}^{n}{W_{\left[t\right]}^{i}}^{2}\right)$

       calculate accuracy *TrainAcc*_[*t*]_ and *TestAcc*_[*t*]_

       calculate growth rate

      *for* ℓ = *Current*_ℓ_

    $\frac{dh_{s}^{l}}{dt}=\frac{1}{E+l}*(\frac{1}{C}+1)*E*(1-\frac{h_{s}^{l}}{C})(h_{s}^{l}-\alpha )$

*//Update the growth rate*

$h_{s[t]}^{l}=h_{s[t-1]}^{l}+\frac{dh_{s}^{l}}{dt}$

          *//Determine if a new unit is grown*.

                if $h_{s[t]}^{l}-h_{s[t-1]}^{l}=1$

*// Determine if the layer has reached the carrying capacity*.

 *if* $h_{s[t]}^{l}<C$

  *// A new hidden unit has been grown*.

                    $S_{\left[t\right]}^{l}=S_{\left[t-1\right]}^{l}+1$

                      *//Random initialization of the new unit’s weights from a uniform distribution ranging from -0*.*1 to 0*.*1*.

 *elif*  $h_{s[t]}^{l}=C$

*Current*_*ℓ*_ = *Current*_*ℓ*_+1 *//Switch to the next layer*.

$S_{\left[t\right]}^{l+1}=1$ //Add the new unit to the next layer.

      if *E*_[*t*]_<*Min*_*E*_ or *t* = *Max*_*t*_ or (*TestAcc*_[*t*]_−*TestAcc*_[*t*−1000]_)<*θ*

*done* = *true*

 *else*

*t* = *t+*1

**Part 2b Learning and Sequential Growing: F-Variant**

*//Only the incoming weights to active layer are updated and others frozen*.

*done = false*

$While\;t<{Max}_{t}\;and\;done=false$

*for iter = 1 to R*

calculate $m_{\left[t\right]}=\beta m_{\left[t-1\right]}+(1-\beta )g_{\left[t\right]}$

*//Only the incoming weights to active layer are updated*.

calculate $W_{\left[t\right]}^{{Current}_{l}}=W_{\left[t-1\right]}^{{Current}_{l}}-\eta (m_{\left[t\right]})$

*end iter*

       calculate loss

$E_{\left[t\right]}=\left(-\frac{1}{M}\sum_{k=1}^{N}\sum_{q=1}^{M}y_{q}^{k}\log{\hat{y}}_{q}^{k}\right)+\left(\frac{\lambda }{2M}\sum_{i=1}^{n}{W_{\left[t\right]}^{i}}^{2}\right)$

       calculate accuracy *TrainAcc*_[*t*]_ and *TestAcc*_[*t*]_

       calculate growth rate

      *for* ℓ = *Current*_ℓ_

    $\frac{dh_{s}^{l}}{dt}=\frac{1}{E+l}*(\frac{1}{C}+1)*E*(1-\frac{h_{s}^{l}}{C})(h_{s}^{l}-\alpha )$

*//Update the growth rate*

$h_{s[t]}^{l}=h_{s[t-1]}^{l}+\frac{dh_{s}^{l}}{dt}$

          *//Determine if a new unit is grown*.

                if $h_{s[t]}^{l}-h_{s[t-1]}^{l}=1$

*// Determine if the layer has reached the carrying capacity*.

 *if*  $h_{s[t]}^{l}<C$

 *// A new hidden unit has been grown*.

                   $S_{\left[t\right]}^{l}=S_{\left[t-1\right]}^{l}+1$

                      *//Random initialization of the new unit’s weights from a uniform distribution ranging from -0*.*1 to 0*.*1*.

 *elif*  $h_{s[t]}^{l}=C$

*Current*_ℓ_ = *Current*_ℓ_+1 *//Switch to the next layer (active layer*).

$S_{\left[t\right]}^{l+1}=1$ //Add the new unit to the next layer.

      if *E*_[*t*]_<*Min*_*E*_ or *t* = *Max*_*t*_ or (*TestAcc*_[*t*]_−*TestAcc*_[*t*−1000]_)<*θ*

*done* = *true*

 *else*

*t* = *t*+*1*

2) DNC growth:

**Part 2a Learning and DNC Growing: UAW-Variant**

*//Update all weights*.

done=false

*t_0_ = 0* *//Time in epochs when the last unit was added*.

$While\;t<{Max}_{t}\;and\;done=false$

*for iter = 1 to R*

calculate *m*_[*t*]_ = *βm*_[*t*−1]_+(1−*β*)*g*_[*t*]_

calculate $W_{\left[t\right]}^{l}=W_{\left[t-1\right]}^{l}-\eta (m_{\left[t\right]})$

*end iter*

       calculate loss

$E_{\left[t\right]}=\left(-\frac{1}{M}\sum_{k=1}^{N}\sum_{q=1}^{M}y_{q}^{k}\log{\hat{y}}_{q}^{k}\right)+\left(\frac{\lambda }{2M}\sum_{i=1}^{n}{W_{\left[t\right]}^{i}}^{2}\right)$

       calculate accuracy *TrainAcc*_[*t*]_ and *TestAcc*_[*t*]_

*//Determine if enough time has passed and the error has stopped changing*.

         *if* $(t-z)\geq t_{0}and\frac{\rho_{\left[t\right]}-\rho_{\left[t-z\right]}}{\rho_{{[t}_{0]}}}<\Delta_{T}$

*// Determine if the layer has reached the carrying capacity*.

  *if*  $S_{\left[t\right]}^{l}<C$

 *// A new hidden unit is added*.

                   $S_{\left[t\right]}^{l}=S_{\left[t-1\right]}^{l}+1$

                      *//Random initialization of the new unit’s weights from a uniform distribution ranging from -0*.*1 to 0*.*1*.

  elif  $S_{\left[t\right]}^{l}=C$

*Current*_ℓ_ = *Current*_ℓ_+1 *//Switch to the next layer*.

$S_{\left[t\right]}^{l+1}=1$ //Add the new unit to the next layer.

*t_0_ = t*

      if *E*_[*t*]_<*Min*_*E*_ or *t* = *Max*_*t*_ or (*TestAcc*_[*t*]_−*TestAcc*_[*t*−1000]_)<*θ*

*done* = *true*

 *else*

*t* = *t*+1

**Part 2b Learning and DNC Growing: F-Variant**

*//Only the incoming weights to active layer are updated and others frozen*.

*t*_0_ = 0 *//Time in epochs when the last unit was added*.

$While\;t<{Max}_{t}\;and\;done=false$

*for iter = 1 to R*

calculate $m_{\left[t\right]}=\beta m_{\left[t-1\right]}+(1-\beta )g_{\left[t\right]}$

*//Only the incoming weights to active layer are updated*.

calculate $W_{\left[t\right]}^{{Current}_{l}}=W_{\left[t-1\right]}^{{Current}_{l}}-\eta (m_{\left[t\right]})$

*end iter*

       calculate loss

$E_{\left[t\right]}=\left(-\frac{1}{M}\sum_{k=1}^{N}\sum_{q=1}^{M}y_{q}^{k}\log{\hat{y}}_{q}^{k}\right)+\left(\frac{\lambda }{2M}\sum_{i=1}^{n}{W_{\left[t\right]}^{i}}^{2}\right)$

       calculate accuracy *TrainAcc*_[*t*]_ and *TestAcc*_[*t*]_

*//Determine if enough time has passed and the error has stopped changing*.

         *if* $(t-z)\geq t_{0}\;and\;\frac{\rho_{\left[t\right]}-\rho_{\left[t-z\right]}}{\rho_{{[t}_{0]}}}<\Delta_{T}$

*// Determine if the layer has reached the carrying capacity*.

  *if*  $S_{\left[t\right]}^{l}<C$

 *// A new hidden unit is added*.

                    $S_{\left[t\right]}^{l}=S_{\left[t-1\right]}^{l}+1$

                      *//Random initialization of the new unit’s weights from a uniform distribution ranging from -0*.*1 to 0*.*1*.

  elif  $S_{\left[t\right]}^{l}=C$

*Current*_ℓ_ = *Current*_ℓ_+1 *//Switch to the next layer (active layer)*.

$S_{\left[t\right]}^{l+1}=1$ //Add the new unit to the next layer.

*t*_0_ = *t*

      if *E*_[*t*]_<*Min*_*E*_ or *t* = *Max*_*t*_ or (*TestAcc*_[*t*]_−*TestAcc*_[*t*−1000]_)<*θ*

*done* = *true*

 *else*

*t* = *t+1*

### Experiments and results

The proposed parallel growing algorithm was compared to the sequential version of our growing algorithm as well as DNC in the MLP on three benchmark classification data sets: the Breast Cancer Wisconsin (Diagnostic; BCW) data set [65], the Wine data set [66], and the Fashion MNIST data set [67]. These data sets vary in the number of features, classes, and instances (see Table 1). For a more detailed comparison, four variants for each approach (Parallel, Sequential, and DNC) were considered. These variants were based on the number of layers pre-initialized; one (First Init) or all (All Init), and the type of weight update; updating all weights (UAW) or just the incoming connections to the active layer and freezing the others (F).

**Table 1:** Data sets.

Data set	Number of features	Number of classes	Number of instances
BCW	30	2	569
Wine	13	3	178
Fashion MNIST	784	10	70,000

The results of the binary classification of the BCW data set are shown in Table 2. With the All Init-UAW variant, all approaches had similar training and testing performances. Slight variations in network size were observed, with the DNC approach growing fewer units than the parallel. With the First Init-UAW variant, all approaches were consistent in terms of size, performance, and training epochs. Given the initialization of only one hidden layer and the task was easier for the network, fewer hidden units were required. The parallel approach behaved similarly to the sequential approach with no additional hidden layers grown. Under the First Init-F variant, similar training and testing performances were observed across all approaches, with DNC having smaller variations. Interestingly, the parallel approach grew three layers and approximately 21 hidden units. Conversely, the sequential and DNC approaches only required one hidden layer and approximately 5 units. Notably, the sequential approach also took considerably fewer training epochs with a smaller standard deviation. Finally, for the All Init-F variant, the parallel approach achieved near-perfect training and testing performances-outperforming both the sequential and DNC approaches, which reached about 60% and 50% respectively. Additionally, both the sequential and DNC approaches reached the maximum number of training epochs, whereas the parallel approach required considerably fewer epochs. On average, the parallel approach, grew smaller (three layers and about 140.6 units) than the sequential (three layers and about 292.9 units) but grew larger than DNC (three layers and about 102.0 units). Notably, due to the window width limitation for DNC (set at 1000 epochs) and the maximum number of hidden units is set at 100 per layer; DNC could not grow units past the first layer.

**Table 2:** Average results of the parallel, sequential, and DNC variants—Breast Cancer Wisconsin.

Approach	Measures				
	Layers	Hidden Units	Training Acc. (%)	Testing Acc. (%)	Epochs taken
All Init-UAW					
Parallel	3	**7.1±3.75** **3.2±1.17** **2.4±0.66**	99.80±0.00	94.79±0.00	6,105.1±1,221.88
Sequential	3	**12.5±6.89** **1.0±0.00** **1.0±0.00**	99.80±0.00	93.90±0.00	7,406.4±1,801.80
DNC	3	**6.7±2.56** **1.0±0.00** **1.0±0.00**	99.80±0.00	96.50±0.00	8,807.8±2,524.43
First Init-UAW					
Parallel	1	1.0±0.00	99.30±0.00	98.20±0.00	2,001.0±0.00
Sequential	1	1.0±0.00	98.70±0.00	100.00±0.00	2,001.0±0.00
DNC	1	1.0±0.00	99.10±0.00	96.50±0.00	2,001.0±0.00
First Init-F					
Parallel	**3**	17.0±27.49 3.0±9.0 0.6±1.8	92.33±4.32	91.31±4.36	6,605.6±5,262.63
Sequential	1	**5.7±1.55**	92.19±4.61	92.91±3.24	**4,003.0±1,266.18**
DNC	1	**4.60±2.37**	93.29±1.77	92.80±1.75	7,206.2±3,519.19
All Init-F					
Parallel	3	86.0±4.02 38.0±5.87 16.6±2.33	**98.11±0.23**	**96.68±0.38**	**16,015.0±895.32**
Sequential	3	100.0±0.00 100.0±0.00 92.9±0.54	63.30±0.00	60.50±0.00	100,000.0±0.00
DNC	3	100.0±0.00 1.0±0.00 1.0±0.00	47.64±12.19	46.66±17.25	100,000.0±0.00

*Note*: Averages were calculated across 10 trials with the std. dev. reported.

The three-class Wine data set showed similar results to the BCW (see Table 3). For the All Init-UAW variant, all approaches had similar average accuracies. However, the sequential approach had a large standard deviation of about 6%. In terms of the average size, DNC was the smallest (24 units), followed by parallel approach (74.9 units), and the sequential approach (101.7 units). Despite not being the smallest, the parallel approach took noticeably fewer training epochs and had less variation. For the First Init-UAW variant, all approaches were almost identical in size, each having approximately one hidden unit. Similar to the BCW dataset, as only one hidden layer was initialized and the task was less complex, fewer hidden units were required. The parallel approach again behaved similar to the sequential approach with no additional hidden layers grown. Performance-wise, all approaches were comparable, with DNC having a larger standard deviation of about 4%. A noteworthy observation is the difference in training epochs. The parallel approach, on average, needed 110 training epochs, while the other approaches needed over 2,000. For the First Init-F variant, the parallel approach grew larger (three hidden layers with an average of 166.8 hidden units) compared to the other approaches that only used one hidden layer (sequential: 23.2 hidden units, and DNC: 5.8 hidden units). Despite the parallel approach having, on average, 2% higher accuracies, it took more than double the training epochs. For the final variant, the All Init-F variant, the parallel and sequential approaches had similar performances. Nevertheless, the sequential method grew to the maximum of 100 units per layer (300 units total) and required an average of over 90,000 training epochs. Comparatively, the parallel approach utilized only 248.3 units with an average of 18,7171 epochs. DNC faced the same limitation, unable to grow beyond the first layer, resulting in poor performances and reaching the maximum number of training epochs.

**Table 3:** Average results of the parallel, sequential, and DNC variants–Wine.

Approach	Measures				
	Layers	Hidden Units	Training Acc. (%)	Testing Acc. (%)	Epochs taken
All Init-UAW					
Parallel	3	47.5±8.35 17.9±3.48 9.5±1.57	100.00±0.00	95.24±1.89	8,890.6±640.40
Sequential	3	97.7±2.97 3.3±3.20 1.0±0.00	96.91±6.46	96.39±6.13	21,999.9±5,145.26
DNC	3	22.8±7.70 1.0±0.00 1.0±0.00	100.0±0.00	99.44±1.18	25,124.1±7,862.15
First Init-UAW					
Parallel	1	1.8±0.40	99.09±0.99	97.20±2.95	110.83±350.46
Sequential	1	1.5±0.50	99.02±1.10	97.20±2.95	2,208.8±224.04
DNC	1	1.0±0.00	95.21±4.21	93.02±4.36	2,101.1±300.3
First Init-F					
Parallel	**3**	91.0±26.0 61.5±24.88 14.3±6.51	94.66±5.01	94.17±5.14	18,117.1±4,575.13
Sequential	1	23.2±12.79	92.48±5.04	91.39±5.91	6,205.2±1,779.42
DNC	1	5.8±2.52	92.05±6.20	92.49±7.31	8,707.7±4,076.61
All Init-F					
Parallel	3	**99.1±0.30** **91.3±2.97** **57.9±4.99**	84.93±7.79	86.39±5.14	**18,717.7±318.12**
Sequential	3	100.0±0.00 100.0±0.00 100.0±0.00	86.26±8.97	88.34±9.86	90,659.9±6,462.79
DNC	3	100.0±0.00 1.0±0.00 1.0±0.00	31.70±3.38	29.76±1.35	100,000.0±0.00

*Note*: Averages were calculated across 10 trials with the std. dev. reported.

The most demanding data set was the ten-class Fashion MNIST (see Table 4). For the All Init-UAW variant, the parallel approach had approximately 40% better average training and testing accuracies compared to both the sequential and DNC. The parallel approach also grew on average substantially smaller (22.6 hidden units) compared to the other approaches (sequential: 259.2 hidden units; DNC 83.0 hidden units) and took only 5,893 epochs to train (about 61,000–94,000 epochs less). For the First Init-UAW variant, both the parallel and sequential approaches had similar sizes, performances, and training epochs on average. Conversely, DNC grew smaller (about three fewer hidden units), had a lower training accuracy (about 5% less), but took significantly more epochs. For the First Init-F variant, the parallel approach had the highest performance. However, on average, the parallel approach also grew significantly larger (three hidden layers with 215 hidden units) compared to the other two approaches (sequential: 51.6 hidden units; DNC 24.8 hidden units). For the final variant, the All Init-F, the parallel approach vastly outperformed the other approaches. The parallel approach achieved approximately 40% higher training and testing accuracies than the sequential approach. It also took fewer training epochs and grew substantially smaller (170 vs. 300 hidden units). The DNC approach encountered the same limitation and could not grow past the first layer. This resulted in poor performances and reaching the maximum number of training epochs.

**Table 4:** Average results of the parallel, sequential, and DNC variants–Fashion MNIST.

Approach	Measures				
	Layers	Hidden Units	Training Acc. (%)	Testing Acc. (%)	Epochs
All Init-UAW					
Parallel	3	**12.9±2.07** **5.8±1.09** **3.9±0.60**	**89.43±1.43**	83.30±0.81	**5,893.7±699.91**
Sequential	3	100.0±0.00 90.5±28.50 68.7±38.68	51.56±41.6	47.59±37.6	70,319.8±30,586.65
DNC	3	81.0±12.21 1.0±0.00 1.0±0.00	14.99±5.15	14.98±5.14	100,000±0.00
First Init-UAW					
Parallel	1	7.2±0.60	88.40±0.86	82.97±0.59	4,903.9±300.30
Sequential	1	7.8±1.08	89.04±0.83	83.23±0.28	5,304.3±458.72
DNC	1	**4.3±0.71**	83.39±4.89	83.39±4.89	**19,121.1±4,914.30**
First Init-F					
Parallel	3	99.5±0.50 82.1±9.68 33.4±7.72	**93.31±2.85**	**87.47±2.37**	14,613.6±917.43
Sequential	1	51.6±12.53	85.08±0.70	83.67±0.68	6,805.8±872.65
DNC	1	**24.8±5.25**	83.82±0.98	81.04±0.91	24,823.8±5,255.01
All Init-F					
Parallel	3	**82.9±8.43** **54.9±9.17** **32.3±5.83**	**94.73±3.95**	**85.98±2.42**	**9,008.0±775.37**
Sequential	3	100.0±0.00 100.0±0.00 100.0±0.00	48.87±9.38	48.63±9.22	100,000.0±0.00
DNC	3	100.0±0.00 1.0±0.00 1.0±0.00	10.00±0.00	10.00±0.00	100,000.0±0.00

*Note*: Averages were calculated across 10 trials with the std. dev. reported.

Regarding the variants, initializing only one hidden layer (First Init) yielded smaller topologies, faster training times, and similar performances across all tasks compared to initializing all the hidden layers (All Init). Similarly, updating all weights (UAW) yielded smaller topologies, faster training times, and similar performances across all tasks compared to using a freezing approach where only the active layer’s weights were updated (F). Overall, the parallel approach rivals its sequential counterpart. With the two and three-class problems, the parallel approach demonstrated comparable performances, often accompanied by the potential for fewer training epochs. However, the improved training efficiency tended to coincide with larger architectures. With the more challenging 10-class problem, the parallel rivaled or outperformed the sequential approaches. The only exception was with the First Init-F variant, where the parallel approach resulted in larger structures and longer training times. Specifically, for both the All Init variants, the parallel approach grew smaller topologies, achieved higher accuracies, and took fewer training epochs. For the First Init-UAW, the parallel approach performed the equivalently to the sequential approach, as no additional hidden layers were needed to perform the task.

## Discussion

This study focused on the challenge of *when a new hidden unit or layer should be added* when using a constructive algorithm. We proposed investigating the effects of growing sequentially or in parallel in a multilayer context. Sequential growth was characterized by only growing one hidden layer at a time. In contrast, parallel growth was characterized by growing all hidden layers simultaneously. To achieve this, we created a modified version of our population dynamics inspired growing algorithm capable of growing in parallel. Several variants of these approaches based on the hidden layer initialization and the training scheme employed were used to ensure a more comprehensive comparison. The sequential and parallel approaches were tested on several benchmark classification tasks. Another sequential growing approach, DNC, was also compared as it used the standard methodology of iteratively adding units based on the error curve flattening within a specific time frame.

The results highlight two scenarios where parallel growing is more beneficial than sequential. The first is realized by comparing to the DNC approach, which iteratively adds units based on the error curve flattening within a specific time frame. The All Init-F variant highlighted the limitation of a constructive approach heavily reliant on fine-tuning hyperparameters. While a smaller trigger slope and a larger window width can lead to smaller topologies, it can prevent the network from sequentially growing multiple layers in a reasonable time frame. In this case, with a maximum of 100,000 training epochs and a higher maximum hidden units per layer, it is impossible to grow depth with DNC if the task requires it. If the hyperparameters are not finely-tuned to the specific task, the resulting architecture can grow larger than the minimal number of units needed or prevent the network from growing beyond the minimal amount [35]. Secondly, for the more challenging 10-class problem, when multiple hidden layers were initialized, the parallel growing approach surpassed the sequential approaches. It offered smaller layer widths, faster training times, and higher performances.

The possibility of the resulting architecture being larger than required for a given task can be considered a limitation of growing in parallel. Increasing network complexity beyond what is required can lead to overfitting [11]. A possible solution to this problem is to create a hybrid growing-pruning approach by further developing the population dynamics inspired approach to include pruning. Including network pruning offers a way to reduce the structural complexity of the network systematically. One way to achieve this would be to have a negative growth rate to decay the neuronal population and obtain an optimal minimal topology. Including a way to prune the network would allow for comparisons to more state-of-the-art approaches, as hybrid methods appear to be more suitable in searching for optimal architectures [16].

In our population dynamics inspired growing algorithm, the global network error is used as a measure of performance feedback that modulates the growth rate of the hidden population. As the error converges toward zero, the growth rate likewise converges toward zero. This property gives rise to the algorithms’ ability to grow near-optimal architectures based on task complexity. One potential issue is the notion of the network error not converging to a minimal value due to noise in the data, resulting in a continuously growing architecture. An alternative might be to use the network bias and variance as a built-in metric for scaling, growing and pruning, such as by calculating network significance [58,59]. Essentially, this could act as a form of early-stopping for the growing process.

The focus of this study was not to obtain the most optimal performance but to investigate the comparison between sequential and parallel growing methods. As such, no preprocessing methods or modifications to the data set were applied. Performing preprocessing techniques or processes such as data augmentation can introduce confounding variables, making meaningful comparisons between models challenging [22]. However, preprocessing allows using more real-world datasets and can lead to faster learning and higher classification accuracies [68]. An interesting extension for comparing sequential and parallel growing would be to implement the constructive algorithm in a CNN to learn larger coloured images, such as the CIFAR-10. CNNs are comprised of convolutional and pooling layers that are responsible for feature extraction, followed by a fully-connected layer that is responsible for classifying the image. Replacing the pre-designed architecture of the fully-connected layer with a constructive algorithm gives rise to a more adaptive CNN. Mohamed et al. [69] replaced the fully-connected layer in a CNN with their cascade-correlation growing deep learning neural network algorithm (CCG-DLNN) to successfully classify lung cancer images. With the success of a sequential growing approach in a CNN, the question arises could a parallel growing approach be beneficial to reduce time spent growing?

## Conclusion

The decision of when to add a new hidden unit or layer is a fundamental challenge for constructive algorithms. It becomes even more complex in the context of multiple hidden layers. The application and testing of parallel growing methods, in general, merit further investigation.

## References

[1] Zhong G, Ling X, Wang LN. From shallow feature learning to deep learning: Benefits from the width and depth of deep architectures. Wiley Interdiscip Rev Data Min Knowl Discov [Internet]. 2019 Jan 1 [cited 2023 Jun 25];9(1):e1255. Available from: https://onlinelibrary-wiley-com.proxy.bib.uottawa.ca/doi/full/10.1002/widm.1255

[2] Hornik K, Stinchcombe M, White H. Multilayer feedforward networks are universal approximators. Neural Networks. 1989 Jan 1;2(5):359–66.

[3] Funahashi KI. On the approximate realization of continuous mappings by neural networks. Neural Networks. 1989 Jan 1;2(3):183–92.

[4] Bianchini M, Scarselli F. On the complexity of neural network classifiers: A comparison between shallow and deep architectures. IEEE Trans Neural Networks Learn Syst. 2014;25(8):1553–65. doi: 10.1109/TNNLS.2013.2293637 25050951

[5] Lecun Y, Bengio Y, Hinton G. Deep learning. Nat 2015 5217553 [Internet]. 2015 May 27 [cited 2022 Jan 25];521(7553):436–44. Available from: https://www.nature.com/articles/nature14539 doi: 10.1038/nature14539 26017442

[6] Eldan R, Shamir O. The power of depth for feedforward neural networks. In: Conference on Learning Theory [Internet]. PMLR: PMLR; 2016 [cited 2022 Jan 25]. p. 907–40. Available from: https://proceedings.mlr.press/v49/eldan16.html

[7] Bengio Y, Courville A, Vincent P. Representation learning: A review and new perspectives. IEEE Trans Pattern Anal Mach Intell. 2013;35(8):1798–828. doi: 10.1109/TPAMI.2013.50 23787338

[8] Craik A, He Y, Contreras-Vidal JL. Deep learning for electroencephalogram (EEG) classification tasks a review. J Neural Eng. 2019;16(3). doi: 10.1088/1741-2552/ab0ab5 30808014

[9] Guo Y, Liu Y, Oerlemans A, Lao S, Wu S, Lew MS. Deep learning for visual understanding: A review. Neurocomputing. 2016 Apr 26;187:27–48.

[10] Zhao ZQ, Zheng P, Xu ST, Wu X. Object detection with deep learning: A review. IEEE Trans Neural Networks Learn Syst. 2019 Nov 1;30(11):3212–32. doi: 10.1109/TNNLS.2018.2876865 30703038

[11] Curteanu S, Cartwright H. Neural networks applied in chemistry. I. Determination of the optimal topology of multilayer perceptron neural networks. J Chemom [Internet]. 2011 Oct 1 [cited 2020 Apr 5];25(10):527–49. Available from: http://doi.wiley.com/10.1002/cem.1401

[12] Islam MM, Sattar MA, Amin MF, Yao X, Murase K. A new adaptive merging and growing algorithm for designing artificial neural networks. IEEE Trans Syst Man, Cybern Part B Cybern. 2009;39(3):705–22. doi: 10.1109/TSMCB.2008.2008724 19203888

[13] Liu D, Chang T-S, Zhang Y. A constructive algorithm for feedforward neural networks with incremental training. IEEE Trans Circuits Syst Fundam Theory Appl. 2002;49(12).

[14] Parekh R, Yang J, Honavar V. Constructive neural-network learning algorithms for pattern classification. IEEE Trans Neural Networks [Internet]. 2000 Mar [cited 2020 Jan 13];11(2):436–51. Available from: http://ieeexplore.ieee.org/document/839013/. doi: 10.1109/72.839013 18249773

[15] Kwok TY, Yeung DY. Constructive algorithms for structure learning in feedforward neural networks for regression problems. IEEE Trans Neural Networks. 1997;8(3):630–45. doi: 10.1109/72.572102 18255666

[16] Zemouri R, Omri N, Fnaiech F, Zerhouni N, Fnaiech N. A new growing pruning deep learning neural network algorithm (GP-DLNN). Neural Comput Appl 2019 3224 [Internet]. 2020 May 24 [cited 2022 May 9];32(24):18143–59. Available from: https://link.springer.com/article/10.1007/s00521-019-04196-8.

[17] Zemouri R, Omri N, Devalland C, Arnould L, Morello B, Zerhouni N, et al. Breast cancer diagnosis based on joint variable selection and Constructive Deep Neural Network. Middle East Conf Biomed Eng MECBME. 2018 Jul 3;2018-March:159–64.

[18] Pérez-Sánchez B, Fontenla-Romero O, Guijarro-Berdiñas B. A review of adaptive online learning for artificial neural networks. Artif Intell Rev [Internet]. 2018 Feb 1 [cited 2021 Feb 21];49(2):281–99. Available from: 10.1007/s10462-016-9526-2.

[19] Khan WA, Chung SH, Awan MU, Wen X. Machine learning facilitated business intelligence (Part II): Neural networks optimization techniques and applications. Ind Manag Data Syst. 2020 Jan 10;120(1):128–63.

[20] Boughrara H, Chtourou M, Ben Amar C, Chen L. Facial expression recognition based on a mlp neural network using constructive training algorithm. Multimed Tools Appl [Internet]. 2016 Jan 1 [cited 2021 Feb 21];75(2):709–31. Available from: https://link.springer.com/article/10.1007/s11042-014-2322-6.

[21] Augasta MG, Kathirvalavakumar T. Pruning algorithms of neural networks—A comparative study. Open Comput Sci [Internet]. 2013 Sep 1 [cited 2021 Apr 6];3(3):105–15. Available from: https://www.degruyter.com/document/doi/10.2478/s13537-013-0109-x/html.

[22] Blalock D, Gonzalez Ortiz JJ, Frankle J, Guttag J. What is the state of neural network pruning? In: Proceedings of Machine Learning and Systems [Internet]. 2020 [cited 2021 Mar 18]. p. 129–46.

[23] Hoefler T, Alistarh D, Ben-Nun T, Dryden N, Peste A. Sparsity in deep learning: Pruning and growth for efficient inference and training in neural networks. J Mach Learn Res [Internet]. 2021 Jan 1 [cited 2023 Jun 27];23:1–124. Available from: https://dl.acm.org/doi/10.5555/3546258.3546499

[24] Reed R. Pruning algorithms—A survey. IEEE Trans Neural Networks. 1993;4(5):740–7. doi: 10.1109/72.248452 18276504

[25] Sharma SK, Chandra P. Constructive neural networks: A review. Int J Eng Sci Technol [Internet]. 2010 [cited 2021 Apr 4];2(12):7847–55. Available from: https://www.researchgate.net/publication/50384469

[26] Thivierge JP, Rivest F, Shultz TR. A dual-phase technique for pruning constructive networks. In: Proceedings of the International Joint Conference on Neural Networks. 2003. p. 559–64.

[27] Tyagi K, Nguyen S, Rawat R, Manry M. Second Order Training and Sizing for the Multilayer Perceptron. Neural Process Lett. 2020;51(1):963–91. Available from: https://link.springer.com/article/10.1007/s11063-019-10116-7

[28] Narasimha PL, Delashmit WH, Manry MT, Li J, Maldonado F. An integrated growing-pruning method for feedforward network training. Neurocomputing. 2008;71(13–15):2831–47.

[29] Mohamed SAEM Mohamed MH, Farghally MF. A new cascade-correlation growing deep learning neural network algorithm. Algorithms 2021, Vol 14, Page 158 [Internet]. 2021 May 19 [cited 2022 Oct 6];14(5):158. Available from: https://www.mdpi.com/1999-4893/14/5/158/htm.

[30] Han HG, Zhang S, Qiao JF. An adaptive growing and pruning algorithm for designing recurrent neural network. Neurocomputing. 2017 Jun 14;242:51–62.

[31] Dai X, Yin H, Jha NK. NeST: A neural network synthesis tool based on a grow-and-prune paradigm. IEEE Trans Comput. 2019 May 2;68(10):1487–97.

[32] Sharma SK, Chandra P. An adaptive slope basic dynamic node creation algorithm for single hidden layer neural networks. In: Proceedings—2010 International Conference on Computational Intelligence and Communication Networks, CICN 2010. 2010. p. 139–44.

[33] Sadreddin A, Sadaoui S. Incremental feature learning using constructive neural networks. In: Proceedings—International Conference on Tools with Artificial Intelligence, ICTAI. IEEE Computer Society; 2021. p. 704–8.

[34] Masmoudi S, Frikha M, Chtourou M, Hamida A Ben. Efficient MLP constructive training algorithm using a neuron recruiting approach for isolated word recognition system. Int J Speech Technol [Internet]. 2011 Mar 27 [cited 2021 Apr 14];14(1):1–10. Available from: https://link-springer-com.proxy.bib.uottawa.ca/article/10.1007/s10772-010-9082-0.

[35] Ash T. Dynamic node creation in backpropagation networks. Conn Sci [Internet]. 1989 Jan 24 [cited 2020 Jan 13];1(4):365–75. Available from: https://www.tandfonline.com/doi/full/10.1080/09540098908915647.

[36] Kamruzzaman SM, Hasan AR, Siddiquee AB, Mazumder MEH. Medical diagnosis using neural network. In: 3rd International Conference on Electrical & Computer Engineering (ICECE) [Internet]. 2004 [cited 2021 Jun 6]. Available from: http://arxiv.org/abs/1009.4572.

[37] Susan S, Dwivedi M. Dynamic growth of hidden-layer neurons using the non-extensive entropy. In: Proceedings—2014 4th International Conference on Communication Systems and Network Technologies, CSNT 2014. IEEE Computer Society; 2014. p. 491–5.

[38] Bertini JR, Do Carmo Nicoletti M. A feedforward constructive neural network algorithm for multiclass tasks based on linear separability. Constr Neural Networks [Internet]. 2009 [cited 2021 Mar 3];258:145–69. Available from: https://link-springer-com.proxy.bib.uottawa.ca/chapter/10.1007/978-3-642-04512-7_8.

[39] Ma L, Khorasani K. Facial expression recognition using constructive feedforward neural networks. IEEE Trans Syst Man, Cybern Part B Cybern. 2004 Jun;34(3):1588–95. doi: 10.1109/tsmcb.2004.825930 15484928

[40] Islam MM, Sattar A, Amin MF, Yao X, Murase K. A new constructive algorithm for architectural and functional adaptation of artificial neural networks. IEEE Trans Syst Man, Cybern Part B Cybern. 2009;39(6):1590–605. doi: 10.1109/TSMCB.2009.2021849 19502131

[41] Subirats JL, Jerez JM, Iván Gómez•, Franco L. Multiclass pattern recognition extension for the new C-Mantec constructive neural network algorithm. Cognit Comput. 2010;2:285–90.

[42] Fontes CH, Embiruçu M. An approach combining a new weight initialization method and constructive algorithm to configure a single feedforward neural network for multi-class classification. Eng Appl Artif Intell. 2021 Nov 1;106:104495.

[43] Wu X, Rózycki P, Wilamowski BM. A hybrid constructive algorithm for single-layer feedforward networks learning. IEEE Trans Neural Networks Learn Syst. 2015 Aug 1;26(8):1659–68. doi: 10.1109/TNNLS.2014.2350957 25216485

[44] Young S, Downs T. CARVE—A constructive algorithm for real-valued examples. IEEE Trans Neural Networks. 1998;9(6):1180–90. doi: 10.1109/72.728361 18255801

[45] Siddiquee AB, Mazumder MEH, Kamruzzaman SM. A constructive algorithm for feedforward neural networks for medical diagnostic reasoning. arXiv Prepr arXiv10094564 [Internet]. 2010 Sep 23 [cited 2023 Jun 23]; Available from: https://arxiv.org/abs/1009.4564v1.

[46] Hernández-Espinosa C, Fernández-Redondo M. On the design of constructive training algorithms for multilayer feedforward. In: Proceedings of the International Joint Conference on Neural Networks. 2002. p. 890–5.

[47] Ma L, Khorasani K. Application of adaptive constructive neural networks to image compression. IEEE Trans Neural Networks. 2002 Sep;13(5):1112–26. doi: 10.1109/TNN.2002.1031943 18244508

[48] Besnard E, Schmitz A, Hefazi H, Shinde R. Constructive neural networks and their application to ship multidisciplinary design optimization. J Sh Res [Internet]. 2007 [cited 2021 Jun 6];51(4):297–312. Available from: https://www.researchgate.net/publication/233667353.

[49] Fahlman SE, Lebiere C. The cascade-correlation learning architecture. Adv Neural Inf Process Syst. 1990;524–32.

[50] Qiao J, Li F, Han H, Li W. Constructive algorithm for fully connected cascade feedforward neural networks. Neurocomputing. 2016 Mar 19;182:154–64.

[51] Shultz TR. A constructive neural-network approach to modeling psychological development. Cogn Dev. 2012 Oct 1;27(4):383–400.

[52] Wu X, Rozycki P, Kolbusz J, Wilamowski BM. Constructive cascade learning algorithm for fully connected networks. In: Artificial Intelligence and Soft Computing: 18th International Conference, ICAISC 2019, Zakopane, Poland [Internet]. Zakopane, Poland: Springer, Cham; 2019 [cited 2022 Jan 23]. p. 236–47. Available from: https://link-springer-com.proxy.bib.uottawa.ca/chapter/10.1007/978-3-030-20912-4_23.

[53] Aran O, Alpaydın E. An incremental neural network construction algorithm for training multilayer perceptrons. In: Artificial Neural Networks and Neural Information Processing Istanbul, Turkey: ICANN/ICONIP. 2003.

[54] Puma-Villanueva WJ, dos Santos EP, Von Zuben FJ. A constructive algorithm to synthesize arbitrarily connected feedforward neural networks. Neurocomputing. 2012 Jan 1;75(1):14–32.

[55] Lehtokangas M. Modelling with constructive backpropagation. Neural Networks. 1999 Jun;12(4–5):707–16. doi: 10.1016/s0893-6080(99)00018-0 12662678

[56] Islam MM, Murase K. A new algorithm to design compact two-hidden-layer artificial neural networks. Neural Networks. 2001 Nov 1;14(9):1265–78. doi: 10.1016/s0893-6080(01)00075-2 11718425

[57] Ma L, Khorasani K. A new strategy for adaptively constructing multilayer feedforward neural networks. Neurocomputing. 2003 Apr 1;51:361–85.

[58] Ashfahani A, Pratama M. Autonomous deep learning: Continual learning approach for dynamic environments. In: Proceedings of the 2019 SIAM International Conference on Data Mining. Society for Industrial and Applied Mathematics Publications; 2019. p. 666–74.

[59] Pratama M, Za’in C, Ashfahani A, Ong YS, Ding W. Automatic construction of multi-layer perceptron network from streaming examples. In: Proceedings of the 28th ACM International Conference on Information and Knowledge Management. Association for Computing Machinery (ACM); 2020. p. 1171–80.

[60] Baluja S, Fahlman SE. Reducing network depth in the cascade-correlation learning architecture [Internet]. School of Computer Science, Carnegie Mellon University; 1994 Oct [cited 2021 Nov 10]. Available from: https://apps.dtic.mil/sti/citations/ADA289352.

[61] Zemouri R. An evolutionary building algorithm for deep neural networks. In: 12th International Workshop on Self-Organizing Maps and Learning Vector Quantization, Clustering and Data Visualization, WSOM 2017—Proceedings. Institute of Electrical and Electronics Engineers Inc.; 2017. p. 1–7.

[62] Guan SU, Li S. Parallel growing and training of neural networks using output parallelism. IEEE Trans Neural Networks. 2002 May;13(3):542–50. doi: 10.1109/TNN.2002.1000123 18244455

[63] Ross M, Berberian N, Chartier S. Should I stay or should I grow? A dynamic self-governed growth for determining hidden layer size in a multilayer perceptron. In: Proceedings of the International Joint Conference on Neural Networks. Institute of Electrical and Electronics Engineers Inc.; 2020.

[64] Sun GQ. Mathematical modeling of population dynamics with Allee effect. Vol. 85, Nonlinear Dynamics. Springer Netherlands; 2016.

[65] Wolberg W, Mangasarian O, Street N, Street W. Breast Cancer Wisconsin (Diagnostic). UCI Machine Learning Repository. 1995.

[66] Aeberhard S, Forina M. Wine. UCI Machine Learning Repository. 1991.

[67] Xiao H, Rasul K, Vollgraf R. Fashion-MNIST: A novel image dataset for benchmarking machine learning algorithms. arXiv Prepr arXiv170807747 [Internet]. 2017 Aug 25 [cited 2023 Jun 21]; Available from: http://arxiv.org/abs/1708.07747.

[68] Asadi R, Kareem SA. Review of feed forward neural network classification preprocessing techniques. In: American Institute of Physics (AIP) Conference Proceedings [Internet]. AIP Publishing; 2014 [cited 2023 Jul 10]. p. 567–73. Available from: /aip/acp/article/1602/1/567/882139/Review-of-feed-forward-neural-network.

[69] Mohamed SAEM, Mohamed MH Farghally MF. Constructive learning of deep neural networks for bigdata analysis. Int J Comput Appl Technol Res. 2020;9(12):311–22.

